# Evaluation of the Robustness of Therapeutic Drug Monitoring Coupled with Bayesian Forecasting of Busulfan with Regard to Inaccurate Documentation

**DOI:** 10.1007/s11095-021-03115-8

**Published:** 2021-10-18

**Authors:** Adrin Dadkhah, Dzenefa Alihodzic, Astrid Broeker, Nicolaus Kröger, Claudia Langebrake, Sebastian G. Wicha

**Affiliations:** 1 Hospital Pharmacy, University Medical Center Hamburg-Eppendorf, Martinistr. 52, 20246 Hamburg, Germany; 2 Dept. of Clinical Pharmacy, Institute of Pharmacy, University of Hamburg, Hamburg, Germany; 3 Department of Stem Cell Transplantation, University Medical Center Hamburg-Eppendorf, Hamburg, Germany

**Keywords:** busulfan, pharmacometrics, sampling, therapeutic drug monitoring

## Abstract

**Background:**

Inaccurate documentation of sampling and infusion times is a potential source of error in personalizing busulfan doses using therapeutic drug monitoring (TDM). Planned times rather than the actual times for sampling and infusion time are often documented.

Therefore, this study aimed to evaluate the robustness of a limited sampling TDM of busulfan with regard to inaccurate documentation.

**Methods:**

A pharmacometric analysis was conducted in NONMEM® 7.4.3 and “R” by performing stochastic simulation and estimation with four, two and one sample(s) per patient on the basis of a one-compartment- (1CMT) and two-compartment (2CMT) population pharmacokinetic model. The dosing regimens consisted of i.v. busulfan (0.8 mg/kg) every 6 h (Q6H) or 3.2 mg/kg every 24 h (Q24H) with a 2 h- and 3 h infusion time, respectively. The relative prediction error (rPE) and relative root-mean-square error (rRmse) were calculated in order to determine the accuracy and precision of the individual AUC estimation.

**Results:**

A noticeable impact on the estimated AUC based on a 1CMT-model was only observed if uncertain documentation reached ± 30 min (1.60% for Q24H and 2.19% for Q6H). Calculated rPEs and rRmse for Q6H indicate a slightly lower level of accuracy and precision when compared to Q24H. Spread of rPE’s and rRmse for the 2CMT-model were wider and higher compared to estimations based on a 1CMT-model.

**Conclusions:**

The estimated AUC was not affected substantially by inaccurate documentation of sampling and infusion time. The calculated rPEs and rRmses of estimated AUC indicate robustness and reliability for TDM of busulfan, even in presence of erroneous records.

**Supplementary Information:**

The online version contains supplementary material available at 10.1007/s11095-021-03115-8.

## Introduction

In allogeneic hematopoietic stem cell transplantation (allo-HSCT), busulfan-based conditioning regimens are commonly used for adult and pediatric patients. For myeloablative conditioning (MAC), there has been extensive research on the matter of determining if there is a correlation between busulfan drug exposure and patient outcome after allo-HSCT. It was shown that on the one hand under-exposure leads to higher risks of relapse and graft rejection, and on the other hand over-exposure results more often in organ toxicity, sinusoidal obstructive syndrome (SOS), acute graft-*versus*-host-disease (aGvHD) and overall higher treatment-related mortality (TRM) (1–4). Moreover, high inter-patient variability in drug exposure, even after intravenous application, makes individual dose selection challenging and therefore using therapeutic drug monitoring (TDM) is advisable in order to ensure that drug exposure is maintained within the narrow therapeutic range (5, 6).

A recent survey by Ruutu *et al*. showed that there is a lack of consistency regarding pharmacokinetic (PK)-guided dose adjustment practices. This not only applies to target parameters but also to the performance of TDM itself (7). Table S1 (Supplementary) provides an overview of published TDM-protocols as performed by different transplant centers, with the main difference lying in the number of blood samples used and the method of estimating the area under the curve (AUC). The two most commonly used methods for AUC-estimation are non-compartmental analysis (NCA) and model-informed precision dosing (MIPD) using Bayesian forecasting. Comparing these methods, it was found that MIPD does not only deliver higher accuracy and precision of estimations but enables the establishment of limited sampling strategies (LSS) as well (2, 4, 8, 9). Moreover, the use of only one method for AUC-estimation and the harmonization of busulfan plasma exposure units (BPEU) facilitates the comparison of TDM-based exposure data and therefore AUC-targets between transplant centers (4, 10).

Besides the deviations between TDM-protocols, there are several other potential sources of error in personalizing busulfan doses – one of them is the inaccurate documentation of sampling and infusion times (5). In clinical practice, planned times rather than the actual times for sampling and infusion rate are often documented, without being fully aware of what these deviations might implicate for AUC estimation. Two previous studies with antibiotics by Van der Meer *et al*. and Alihodzic *et al*. found that inaccurate documentation may significantly affect individual Bayesian estimation (11, 12). For instance, the accuracy of the individual estimation of central volume of distribution (V1) for meropenem can decrease by 24.6% if documented sampling times deviate 15 min from actual sampling times (12).

However, there is no data regarding the impact of erroneous records on TDM of busulfan.

Therefore, this study aims to evaluate the robustness of a limited sampling TDM coupled with Bayesian forecasting of busulfan with regard to inaccurate documentation of sampling and infusion times.

## Materials and Methods

### Study Design and Dataset Generation in „R “

A pharmacometric study was conducted in NONMEM® (version 7.4.3, ICON, Gaithersburg, MD, USA) and “R” by performing stochastic simulation and estimation (SSE) of 1000 clinical trial simulations comprising 100 patients each on the basis of two different published population pharmacokinetic (popPK) models (13, 14). The dosing regimens consisted of i.v. busulfan with 0.8 mg/kg every 6 h (Q6H) or 3.2 mg/kg every 24 h (Q24H) with a 2 h and 3 h infusion rate, respectively. Simulations and estimations were based on the scenarios of four, two or only one blood sample per patient for both Q24H and Q6H. The sampling times for each sampling design were determined beforehand by performing simulation and estimation, assuring that the sampling designs were unbiased themselves. As a starting point we used our local TDM-sampling design for Q24H, consisting of four blood samples at 3.08, 4, 5 and 6.5 h after start of infusion. The sampling times for Q6H and subsequently for the limited sampling designs for Q24H and Q6H were derived from our local TDM-protocol and examined to be unbiased as well. An overview of the utilized sampling times is shown in Table I.

**Table I:** Utilized Sampling Times after Start of Infusion

Number of samples	Q24H [h]	Q6H [h]
4	3.08, 4, 5, 6.5	2.08, 3, 4, 5.5
2	3.5, 6.5	2.5, 5.5
1	6.5	5.5

### Population Pharmacokinetic Models

The popPK models of Choi *et al*. (13) and McCune *et al*. (14) served as a basis for this pharmacometric study. The one-compartment (1CMT) model of Choi *et al*. (13) was developed based on 101 busulfan blood samples from 36 adult patients undergoing allo-HSCT. While 15 patients received 16 doses of i.v. busulfan (0.8 mg/kg) every 6 h with a 2 h infusion rate (Q6H), the other 21 patients were dosed with four busulfan infusions with 3.2 mg/kg every 24 h with a 3 h infusion rate (Q24H). Blood samples were drawn at pre-infusion, 2, 4, and 6 h after beginning of the first infusion, regardless of the dosing regimen. In comparison, the popPK model of McCune *et al*. (14) was best described as a two-compartment (2CMT) model. It was built on 12,380 busulfan plasma concentrations obtained from 1387 patients with a Q6H- and 166 patients with a Q24H regimen.

### Simulation and Estimation in NONMEM

Stochastic simulation and estimation was conducted in NONMEM® using the final model parameter estimates of the popPK model of Choi *et al*. (13) for a 1CMT-model and from the model of McCune *et al*. (14) for a 2CMT-model. In order to evaluate the impact of inaccurate documentation, uncertainties, hence the deviation from actual and planned sampling and infusion times, were randomly added in R to the planned sampling and infusion times with a standard deviation of ± 5 to ± 30 min before simulation. Adding uncertainties was achieved by using the “rnorm"-function as a first step, which generates a vector of normally distributed random numbers, followed by the exclusion and resampling of time points before application of the infusion. Hence, the uncertainty was individually added for each time point around the planned time. For the 1CMT-model, patient characteristics for the covariates total body weight (TBW) and GSTA1 status were randomly added by using lognormal distribution (mean 57.3 kg, SD 0.12) for TBW and the “rbinom”-function with a probability of 29% for a GSTA1 polymorphism, respectively. For the 2CMT-model, to avoid generation of artifacts, we used the typical covariates reported for this model, since the covariates which were included in the model were partly correlated.

Subsequently, 1000 virtual clinical trials containing 100 patients each were simulated by including sampling and infusion times, which correspond to the accurately documented times. Following, estimation was done by using both accurately documented and planned times for sampling and for infusion time, as well as for the combination of both.

### Impact on Estimated AUC

The results of the stochastic simulation and estimation were evaluated by comparing the estimated patient-individual area under the curve (AUC) from both accurate and planned sampling and infusion times to the true individual AUC from the stochastic simulation step. AUC was calculated by integration of the concentration over time, using the individual estimates for each patient.

In order to determine accuracy and precision of the individual AUC estimation and for better comparison between the two dosing regimens, the relative prediction error (rPE) and the relative root-mean-square error (rRmse) were calculated as follows:

$$rPE \left[\%\right]= \frac{ {estimated}_{i}-{true}_{i}}{{true}_{i}} x 100$$

$$rRmse\left[\%\right]=\sqrt{\frac{1}{N} {\sum }_{1}^{i}\frac{{\left(estimatedi-truei\right)}^{2} }{{truei}^{2}}\times 100}$$

with ‘estimated_i_’ or ‘true_i_’ representing the i’th estimated or true individual AUC.

## Results

### Impact of Inaccurate Documentation of Sampling and Infusion Time for Q24H

**Fig. 1:**
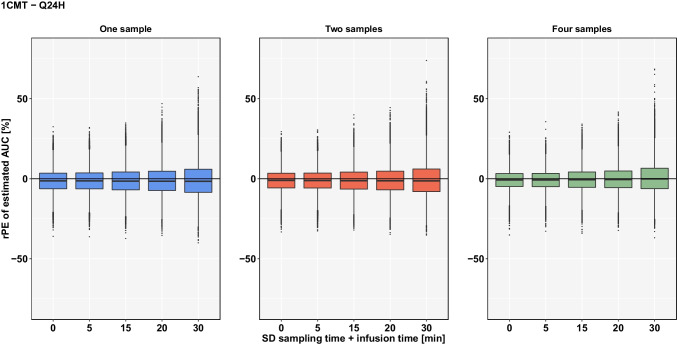
rPE of estimated AUC based on a **1CMT-model** by uncertainty in sampling and infusion time (SD ± 5 min to ± 30 min) if TDM coupled with Bayesian forecasting within **Q24H** is conducted with 1 sample (blue), 2 samples (orange) or 4 samples (green) using planned sampling times.

For the 1-CMT-model, the estimated AUC was not substantially affected by inaccurate documentation of sampling and infusion time for the Q24H scenario (Fig. 1). When only one sample was used for AUC estimation and there was an uncertainty of 30 min both in sampling and infusion time, the median rPE of estimated AUC was -1.60% indicating only a minor bias. In comparison, with 0 min uncertainty, the median rPE was -1.31%. However, at SD ± 30 min the spread of the rPE’s (2.5^th^/97.5^th^ percentile) was distinctly higher (-20.12/22.91%) compared to the minor inaccuracies in documentation of sampling and infusion time at SD ± 5 min (-15.84/13.26%). An overview of the results is provided in Table II.

**Table II:** rPE and rrmse of Estimated AUC for **Q24H**

SD sampling time + SD infusion time [min]		No. of samples—1CMT			No. of samples—2CMT		
		1	2	4	1	2	4
	**rPE** median [%]	-1.31	-1.05	-0.73	-0.51	-1.11	-0.88
	**spread** (2.5^th^/97.5^th^) [%]	28.49	27.58	25.34	56.79	49.20	41.68
0		(-15.61/12.88)	(-15.39/12.19)	(-14.09/11.25)	(-24.11/32.68)	(-23.12/26.08)	(-20.55/21.31)
	**rRmse** [%]	7.41	7.06	6.39	14.54	12.60	10.66
	**rPE** median [%]	-1.33	-1.11	-0.75	-0.56	-1.16	-0.79
	**spread** (2.5^th^/97.5^th^) [%]	29.1	28.01	25.71	56.83	49.90	42.08
5		(-15.84/13.26)	(-15.44/12.57)	(-14.09/11.62)	(-24.02/32.81)	(-23.45/26.45)	(-20.81/21.27)
	**rRmse** [%]	7.53	7.20	6.48	14.61	12.73	10.74
	**rPE** median [%]	-1.47	-1.21	-0.59	-0.34	-0.92	-1.19
	**spread** (2.5^th^/97.5^th^) [%]	32.68	31.78	28.99	57.76	51.40	44.49
15		(-16.85/15.83)	(-16.41/15.37)	(-14.64/14.35)	(-24.25/33.51)	(-23.56/27.84)	(-22.18/22.31)
	**rRmse** [%]	8.43	8.12	7.33	14.82	13.14	11.22
	**rPE** median [%]	-1.55	-1.29	-0.50	-0.25	-0.97	-1.32
	**spread** (2.5^th^/97.5^th^) [%]	35.69	34.62	31.47	58.74	52.79	45.09
20		(-17.73/17.96)	(-17.17/17.45)	(-15.23/16.24)	(-24.73/34.01)	(-24.18/28.61)	(-22.20/22.89)
	**rRmse** [%]	9.13	8.86	7.96	15.07	13.50	11.48
	**rPE** median [%]	-1.60	-1.35	-0.11	-0.15	-0.17	-1.13
	**spread** (2.5^th^/97.5^th^) [%]	43.03	42.06	38.62	61.36	56.16	48.13
30		(-20.12/22.91)	(-19.30/22.76)	(-16.71/21.91)	(-25.38/35.98)	(-25.20/30.96)	(-22.62/25.51)
	**rRmse** [%]	11.03	10.78	9.85	15.60	14.32	12.28

As for the different sampling schedules, the spread of rPE’s at SD ± 5 min increases with the decreasing number of sampling times. Similar results were found for the imprecision, where a decreasing number of samples resulted in a higher rRmse. If four samples were used, an uncertainty of 30 min in sampling and infusion time resulted in a rRmse of 9.85%. In comparison, if TDM was conducted with two samples, rRmse slightly increased to 10.78%. Even if only one sample was used for the estimation of AUC, rRmse did not exceed 11.03%.

Thus, as expected, estimations are slightly more accurate and precise if TDM is conducted with four blood samples compared to two or a single blood sample.

**Fig. 2:**
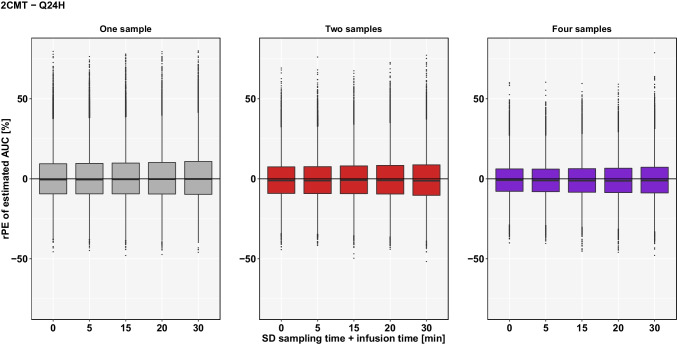
rPE of estimated AUC based on a **2CMT-model** by uncertainty in sampling time and infusion time (SD ± 5 min to ± 30 min) if TDM coupled with Bayesian forecasting within **Q24H** is conducted with 1 sample (grey), 2 samples (red) or 4 samples (purple) using planned sampling times.

For the 2CMT-model, both the spread of rPE’s and the rRmse are considerably wider and higher, respectively. If two samples were used and there was a deviation of 30 min in sampling and infusion times, the spread of rPE’s increased to 56.16% (25.20/30.96%), which indicates a lower level of accuracy for the 2CMT-estimations. The same trend can be observed for the precision of AUC-estimations. For the same scenario, the rRmse of estimated AUC was 14.32% for the 2CMT-model compared to 10.78% rRmse for the 1CMT-model. An overview of the results of the estimated AUC based on a 2CMT-model is shown in Fig. 2 and Table II.

### Impact of Inaccurate Documentation of Sampling and Infusion Time for Q6H

**Fig. 3:**
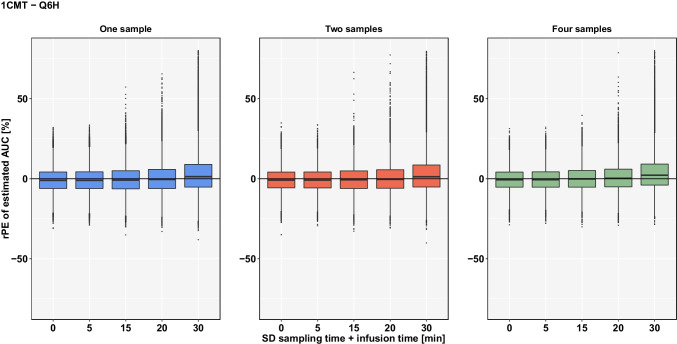
rPE of estimated AUC based on a **1CMT-model** by uncertainty in sampling and infusion time (SD ± 5 min to ± 30 min) if TDM coupled with Bayesian forecasting within **Q6H** is conducted with 1 sample (blue), 2 samples (orange) or 4 samples (green) using planned sampling times.

For the 1CMT-model, the spread of rPE’s of the estimated AUC for 1-point-sampling at an uncertainty of ± 30 min was 49.09%, which is a substantial increase compared to SD ± 0 (30.17%). The median rPE was 1.35% indicating almost no bias. Figure 3 shows that the general trend of increasing spread of rPE’s with increasing uncertainty in documentation is confirmed for Q6H as well.

Also, the spread of rPE’s for Q6H indicate a slightly lower accuracy for each sampling schedule when compared to Q24H. At 0 min uncertainty, for Q6H the spread of rPE’s was 27.41% (-13.86/13.55%) when AUC was estimated with 4 samples, whereas for Q24H the spread was smaller with 25.34%

(-14.09/11.25%). A further comparison at SD ± 20 min with 2-point-sampling shows a spread of rPE’s of 34.62% (-17.17/17.45%) for Q24H and 34.82% (-15.75/19.07%) for Q6H.

Nevertheless, even with increasing spread of rPE’s, a noticeable impact on the median rPE of estimated AUC can only be observed if uncertain documentation of sampling and infusion times reaches ± 30 min (2.19%). The calculated rRmse for Q6H indicates a slightly lower level of precision for each sampling schedule when compared to Q24H (Table II). Again, the tendency of increasing imprecision with decreasing number of samples applies to Q6H, in which the rRmse for the estimated AUC does not exceed 13.26%.

Regarding the impact of either uncertainty in sampling time or infusion time independently (Supplementary Figures S1-S4), for both Q24H and Q6H higher prediction errors occurred if the documented sampling time was erroneous. For instance, within Q24H with two samples, the spread of rPE’s was 27.95% (-15.63/12.32%) if the documented infusion time deviated 30 min from the actual infusion time. For the same scenario, the spread of wrongly estimated AUC increased if the sampling time had an uncertainty of 30 min instead (42.18%).

**Fig. 4:**
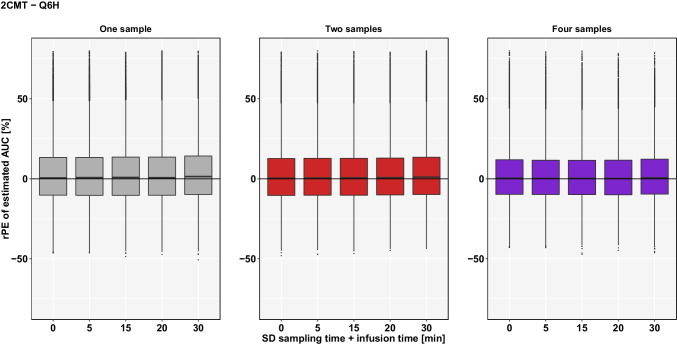
rPE of estimated AUC based on a **2CMT-model** by uncertainty in sampling time and infusion time (SD ± 5 min to ± 30 min) if TDM coupled with Bayesian forecasting within **Q6H** is conducted with 1 sample (grey), 2 samples (red) or 4 samples (purple) using planned sampling times.

For the 2CMT-model, there was also an increase in spread of rPE’s and the rRmse compared to AUC-estimations based on a 1CMT-model. For instance, if two samples were used, an uncertainty of 30 min in sampling and infusion time resulted in a spread of rPE’s of 69.10% and rRmse of 18.06% compared to 47.37% and 12.82% for the 1CMT-model, respectively. An overview of the results for the 2CMT-model can be found in Table III and Fig. 4.

**Table III:** rPE and rrmse of Estimated AUC for **Q6H**

SD sampling time + SD infusion time [min]		No. of samples—1CMT			No. of samples—2CMT		
		1	2	4	1	2	4
	**rPE** median [%]	-1.03	-0.87	-0.63	0.54	0.29	0.34
	**spread** (2.5^th^/97.5^th^) [%]	30.17	28.96	27.41	70.77	68.14	64.14
0		(-15.27/14.90)	(-14.74/14.22)	(-13.86/13.55)	(-27.45/43.32)	(-26.82/41.32)	(-25.44/38.70)
	**rRmse** [%]	7.71	7.42	7.01	18.32	17.71	16.56
	**rPE** median [%]	-1.06	-0.83	-0.59	0.75	0.39	0.18
	**spread** (2.5^th^/97.5^th^) [%]	30.41	29.51	27.64	71.21	68.41	63.85
5		(-15.38/15.03)	(-15.09/14.42)	(-13.84/13.80)	(-27.21/44.00)	(-26.73/41.68)	(-25.58/38.27)
	**rRmse** [%]	7.81	7.51	7.09	18.48	17.68	16.50
	**rPE** median [%]	-0.83	-0.69	-0.16	0.87	0.53	0.20
	**spread** (2.5^th^/97.5^th^) [%]	33.12	32.12	29.91	71.52	68.71	63.96
15		(-16.11/17.01)	(-15.71/16.41)	(-14.14/15.77)	(-27.31/44.21)	(-26.86/41.85)	(-25.74/38.22)
	**rRmse** [%]	8.48	8.23	7.66	18.49	17.77	16.50
	**rPE** median [%]	-0.44	-0.37	0.28	0.71	0.68	0.15
	**spread** (2.5^th^/97.5^th^) [%]	36.40	34.82	32.47	71.22	68.29	64.77
20		(-16.41/19.99)	(-15.75/19.07)	(-14.38/18.09)	(-27.32/43.90)	(-26.70/41.59)	(-25.78/38.99)
	**rRmse** [%]	9.30	8.91	8.35	18.40	17.79	16.67
	**rPE** median [%]	1.35	1.28	2.19	1.40	1.02	0.59
	**spread** (2.5^th^/97.5^th^) [%]	49.09	47.37	43.20	72.05	69.10	73.69
30		(-16.30/32.79)	(-16.05/31.32)	(-14.15/29.05)	(-27.02/45.03)	(-26.45/42.65)	(-25.60/39.04)
	**rRmse** [%]	13.26	12.82	11.90	18.82	18.06	16.76

## Discussion

This is the first study to evaluate and confirm the robustness of TDM coupled with Bayesian forecasting of busulfan with regard to inaccurate documentation of sampling and infusion times in a simulation study. Furthermore, it shows that TDM with a limited sampling strategy is reliable even if erroneous records, which in clinical practice presumably tend to occur, are taken into account.

The data of our pharmacometric study show that for both Q24H and Q6H notable decreases in accuracy and precision only occur if documented sampling and infusion times deviate from actual times as far as 30 min, which in practice can be considered as rather exaggerated. The fact that for no sampling schedule the median rPE of estimated AUC exceeded 5% demonstrates robustness and a sufficient accuracy for personalized dosing of busulfan.

A comparison of the dosing regimens Q24H and Q6H in terms of accuracy of the estimated AUC shows a slightly higher impact on Q6H. This might be due to the fact that with an infusion time of 2 h compared to 3 h within Q24H, for instance 20 min of uncertainty in infusion time is relatively higher for Q6H (17% for Q6H *vs*. 11% for Q24H).

In general, we can see that the accuracy and precision of AUC estimations decreases if only two samples or even one sample are used for the estimation. However, in some cases, for instance for 4-point-sampling within Q24H (Fig. 1), we can observe the median rPE tending towards zero with increasing uncertainty. This of course does not mean that TDM is more accurate with higher uncertainty but is due to the fact that uncertainties are added randomly with the possibility of over- or underestimating the AUC and in this case at some point the estimations evened out in the process of 1000 virtual estimations.

On an overall basis, the outcome of this study was hardly predictable for two reasons: on the one hand, busulfan has a rather short elimination half-life of 2—3 h, hence an uncertainty in sampling time of 30 min or in other words 17—25% of its half-life elimination might suggest the impact on estimating the AUC would be significant, since time is a main factor. On the other hand, most popPK models for busulfan are 1-compartment models, thus it is assumed that the pharmacokinetic behavior of busulfan is not as complex as i.e. of vancomycin, which is often described in 2-compartmental models (15) and compared to busulfan, central volume of distribution (V1), distribution clearance (Q) and peripheral volume of distribution (V2) of meropenem and caspofungin were considerably affected by inaccurate documentation of sampling and infusion times due to the more complex model structure (12). However, even if the popPK-model of busulfan is described as a 2CMT-model (14) and therefore accuracy and precision of the individually estimated AUC is considerably lower in comparison to AUC-estimations based on a 1CMT-model, the calculated rPE’s and rRmse’s (Table II + III) indicate robustness for TDM of busulfan independent of the model complexity. Even though the spread of rPE’s is 70.77% if only one sample was used within Q6H and therefore the estimated AUC should be considered carefully, unbiased estimation of AUC is generally possible even with a single sample since imprecision (expressed as rRmse) does not exceed 18.82%. Interestingly, the impact of inaccurate documentation on AUC-estimations based on a 2CMT-model is less noticeable.

Generally, estimations based on popPK models using Bayesian forecasting are superior to the NCA in terms of accuracy and precision (4, 8, 9). Therefore, we are convinced that MIPD of busulfan will become standard operating procedure in the majority of centers and consequently the evaluation of its robustness regarding the challenges of daily clinical practice was already overdue.

In addition, performing TDM coupled with Bayesian forecasting enables the use of limited sampling strategies (2). Compared to the NCA, where most TDM protocols consist of five blood samples (8, 16, 17), MIPD can be performed with as little as one blood sample, as our data confirm, even with inaccurate documentation.

However, for a reliable TDM we recommend a sampling schedule with at least two blood samples in case one of them cannot be used due to mishandling in processing. Reducing the number of samples needed not only lessens stress for patients due to fewer blood draws, but also decreases cost by requiring fewer bioanalytical assays. According to Palmer *et al*. the cost for bioanalysis per sample ranges from $125-$225, which depending on the number of TDMs performed per year, could mean a substantial reduction of cost if fewer samples are used in clinical practice (2).

Nevertheless, there are few limitations that need to be kept in mind. Even though our results show that MIPD based AUC estimation itself is robust with regard to inaccurate documentation, the popPK model that TDM coupled with Bayesian forecasting is based on, heavily relies on accurately documented data (12). In some cases, model building is conducted with rich sampling schedules that were specifically designed for the purpose of pharmacokinetic analysis (18). In other cases, data from clinical routine TDM is used for model development (19) and therefore it might have been the case that awareness for accurate documentation was not given.

Furthermore, the estimated AUC is based on sampling schedules that we initially examined to be unbiased and therefore the results of our study only apply to the sampling times that are presented in Table I.

In summary, this work shows that TDM coupled with Bayesian forecasting of busulfan is robust with regard to inaccurate documentation and can be conducted with limited sampling. Moreover, confirming robustness for MIPD of busulfan is a further step towards medication safety in HSCT patients.

However, the importance of accurate documentation should not be disregarded, as it is essential to Bayesian forecasting and therefore essential to personalizing busulfan doses.

## Supplementary Information

Below is the link to the electronic supplementary material.

Supplementary file1 (PDF 545 kb)

## Data Availability

The datasets generated for this study are available on request to the corresponding author.
