# Supplementary material for: Evaluation of the Robustness of Therapeutic Drug Monitoring Coupled with Bayesian Forecasting of Busulfan with Regard to Inaccurate Documentation
Source: Pharm Res. 2021 Oct 18;38(10):1721–9. doi: 10.1007/s11095-021-03115-8 (PMC8602150; doi:10.1007/s11095-021-03115-8)
Supplement: Supplementary file 1 — Supplementary file1 (PDF 545 kb) [file 11095_2021_3115_MOESM1_ESM.pdf]

Supplementary Information

# Evaluation of the robustness of therapeutic drug monitoring coupled with Bayesian forecasting of busulfan with regard to inaccurate documentation

*Influence of inaccurate documentation on model-informed precision dosing of busulfan*

Adrin Dadkhah[1,3], Dzenefa Alihodzic[1], Astrid Broeker[3], Nicolaus Kröger[2], Claudia Langebrake[1,2], Sebastian G. Wicha[3]

**Pharmaceutical Research**

**Table S1** Overview of published sampling schedules (sampling times after start of infusion) and corresponding TDM-methods: NCA: Noncompartmental Analysis; Bayesian: Bayesian Forecasting/popPK model-based; 1-CA: 1-Compartmental Analysis; 2-CA: 2-Compartmental Analysis

| | Q24H | | Q6H | | |
|---|---|---|---|---|---|
| Source | No. of samples | Sampling times [h] | No. of samples | Sampling times [h] | PK analysis |
| Salman et al. 2017 | 5 | 0, 3, 6, 12, 18, 24 | 5 | 0, 2, 3, 4, 6 | NCA |
| de Castro et al. 2015 | NA | NA | 5 | 0.5, 2.25, 3, 4, 6 | NCA |
| DoseMe | NA | NA | 2 | 2.5, 6 | Bayesian |
| Salinger et al. 2010 | 5 | 3, 3.25, 4.5, 6, 8 | NA | NA | NCA |
| Sandström et al. 2001 | NA | NA | 3 | 2, 4, 5 | Bayesian |
| Perkins et al. 2011 | 5 | 2, 3.25, 4, 6, 9 | NA | NA | 1-CA |
| Lee et al. 2012 | 4 | 3, 4, 5, 7 | NA | NA | 2-CA |
| Shukla et al. 2020 | NA | NA | NA | NA | Bayesian |
| Dupuis et al. 2008 | NA | NA | 3 | 4, 5, 6 | 1-CA |
| Nguyen et al. 2006 | NA | NA | 2 | 2.25, 6 | Bayesian |
| Long-Boyle et al. 2016 | NA | NA | 5 | 0, 2.25, 2.5, 4, 6 | Bayesian |
| Teitelbaum et al. 2020 | NA | NA | 4 | 0, 2, 3, 4 | NCA |

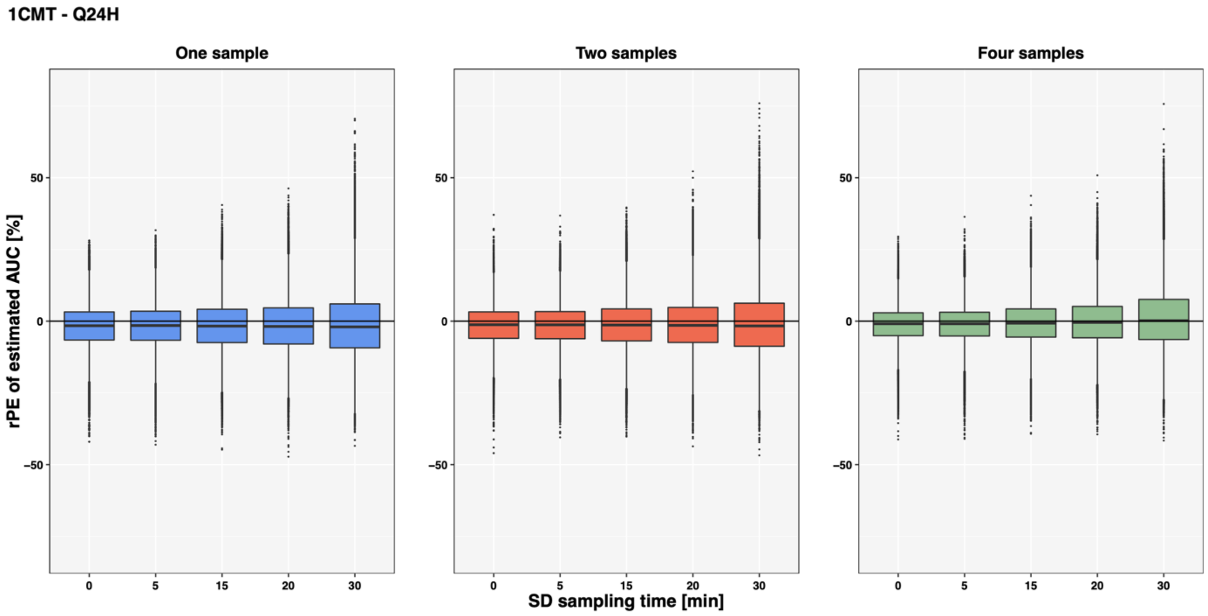


**Fig. S1** rPE of estimated AUC based on a **1CMT-model** by **uncertainty in sampling time** (SD ± 5 min to ± 30 min) if TDM coupled with Bayesian forecasting within **Q24H** is conducted with 1 sample (blue), 2 samples (orange) or 4 samples (green) using planned sampling times

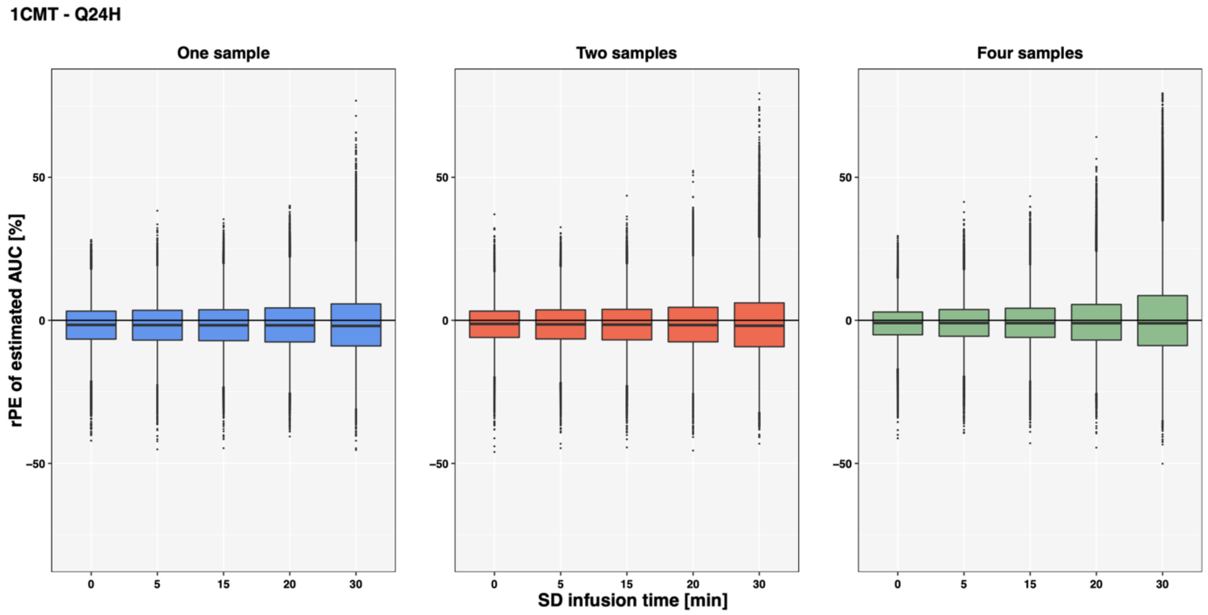


**Fig. S2** rPE of estimated AUC based on a **1CMT-model** by **uncertainty in infusion time** (SD ± 5 min to ± 30 min) if TDM coupled with Bayesian forecasting within **Q24H** is conducted with 1 sample (blue), 2 samples (orange) or 4 samples (green) using planned sampling times.

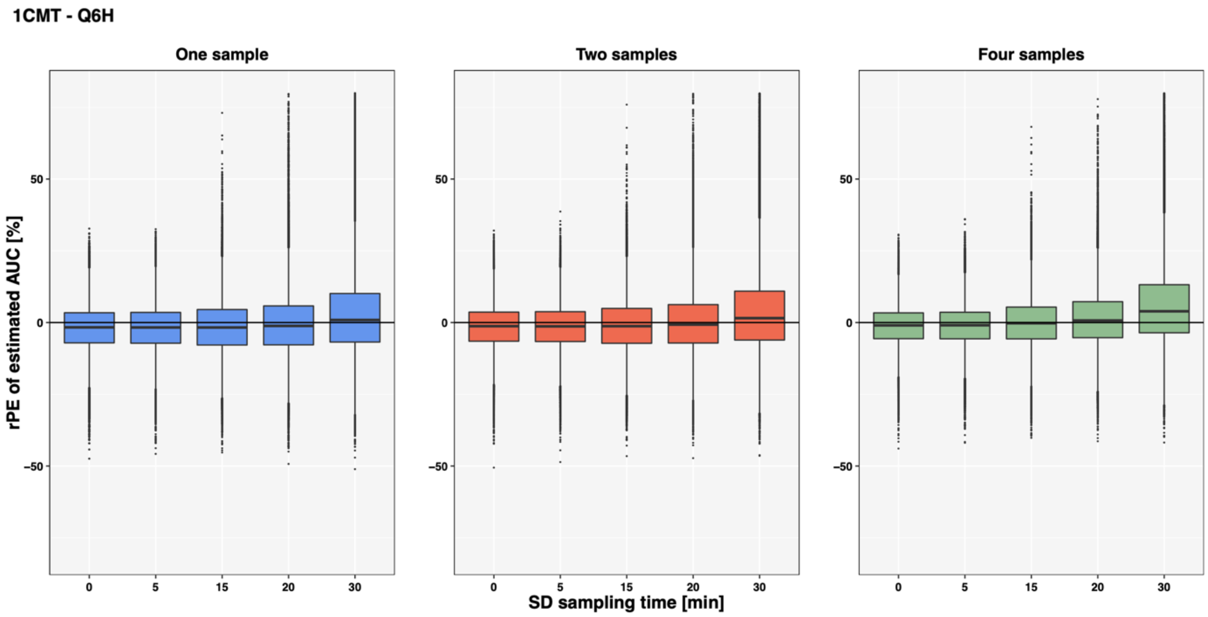


**Fig. S3** rPE of estimated AUC based on a **1CMT-model** by **uncertainty in sampling time** (SD ± 5 min to ± 30 min) if TDM coupled with Bayesian forecasting within **Q6H** is conducted with 1 sample (blue), 2 samples (orange) or 4 samples (green) using planned sampling times.

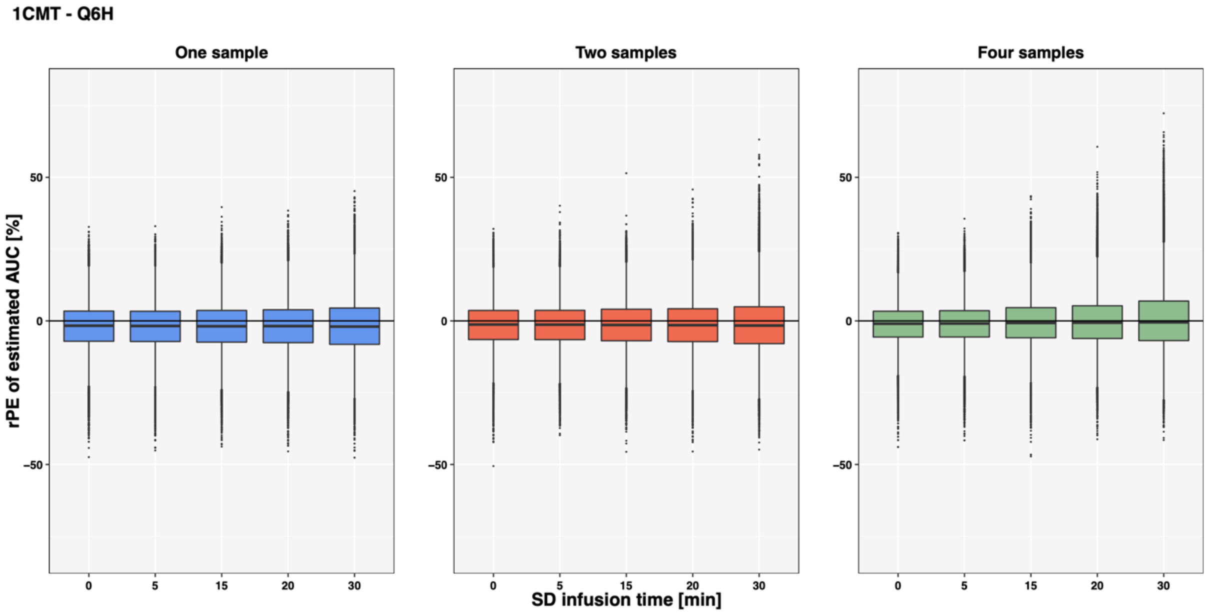


**Fig S4** rPE of estimated AUC based on a **1CMT-model** by **uncertainty in infusion time** (SD ± 5 min to ± 30 min) if TDM coupled with Bayesian forecasting within **Q6H** is conducted with 1 sample (blue), 2 samples (orange) or 4 samples (green) using planned sampling times.
